# A Vexing Diagnosis: ENT Presentation of Vacuoles, E1 Enzyme, X-linked, Autoinflammatory, Somatic (VEXAS) Syndrome

**DOI:** 10.7759/cureus.97628

**Published:** 2025-11-24

**Authors:** Courtney Wilson, James Nightingale, Hershil Khatri, Bernard Whitfield

**Affiliations:** 1 College of Medicine, Princess Alexandra Hospital, Brisbane, AUS; 2 Otolaryngology - Head and Neck Surgery, Toowoomba Hospital, Toowoomba, AUS; 3 General Surgery, Ipswich Hospital, Ipswich, AUS; 4 Otolaryngology - Head and Neck Surgery, Logan Hospital, Brisbane, AUS

**Keywords:** auto-inflammatory syndromes, deep neck space, ent, longus colli calcific tendonitis, vexas syndrome

## Abstract

We report a novel manifestation of Vacuoles, E1 enzyme, X-linked, autoinflammatory, somatic (VEXAS) syndrome characterized by deep neck space involvement in a 75-year-old male. A diagnosis of VEXAS syndrome was established through genetic testing, which revealed variants in the UBA1 and DNMT3A genes following a collaborative multidisciplinary approach. This case highlights a novel syndrome for the ENT surgeon and represents the first case report of deep neck space involvement. It demonstrates the heterogeneity of ENT and soft tissue manifestations of the disease and the complex clinical presentation of this emergent autoinflammatory condition.

## Introduction

Vacuoles, E1 enzyme, X-linked, autoinflammatory, somatic (VEXAS) syndrome is a late-onset autoinflammatory condition resulting from somatic mutations in the UBA1 gene in hematopoietic stem and progenitor cells [1,2]. First identified in 2020, VEXAS syndrome presents with a wide range of systemic inflammatory symptoms and hematological features [3]. While the full spectrum of ENT manifestations has yet to be established, it is reportedly associated with aural and nasal chondritis [1]. We present a case of VEXAS syndrome with atypical deep neck space involvement, not previously described in the literature. This article introduces VEXAS syndrome to the ENT surgeon and aims to promote collaboration across hematology and medical multidisciplinary teams.

## Case presentation

A 75-year-old male presented to a regional emergency department with a three-month history of fever, left neck pain, and unintentional 20 kg weight loss. His medical history included gout, heart failure, type 2 diabetes mellitus, and rheumatoid arthritis. He was a lifelong nonsmoker. He had a stable, long-standing, small, non-tender left neck lump. He denied dysphonia, dysphagia, and odynophagia. He had no sinonasal or otological symptoms. On examination, there was a superficial, mobile, non-tender mass in the left level 2/3 neck and no additional palpable cervical lymphadenopathy. Otoscopy and anterior rhinoscopy were unremarkable.

**Figure 1:**
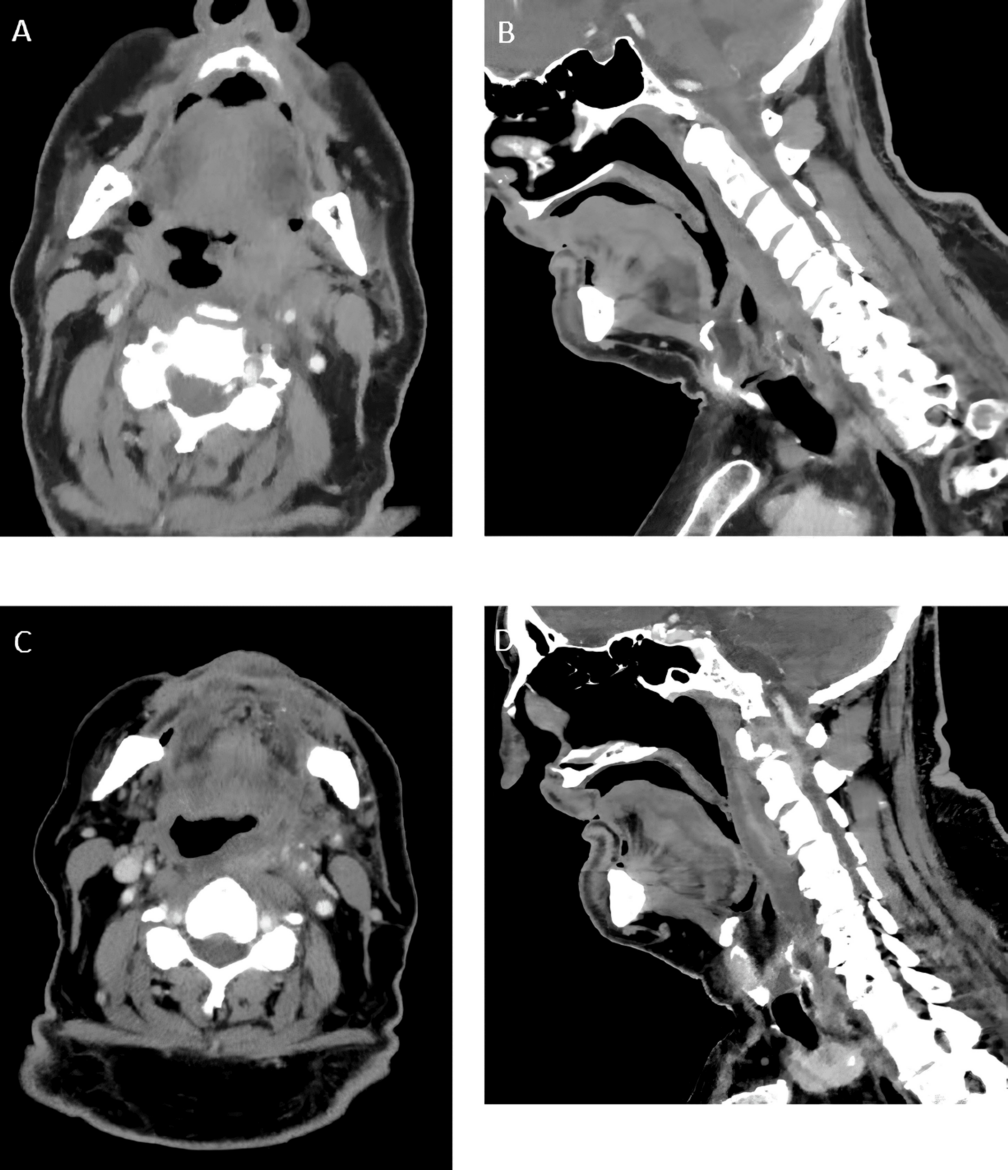
Serial CT images in axial (A, C) and sagittal (B, D) planes demonstrating progressive para- and retropharyngeal effacement. Initial CT (A, B) suggested an infiltrative mass. Subsequent imaging (C, D) demonstrated a more well-defined linear region of density extending from C2 to C4, suggestive of calcific tendinitis of the longus colli. CT, computed tomography

**Figure 2:**
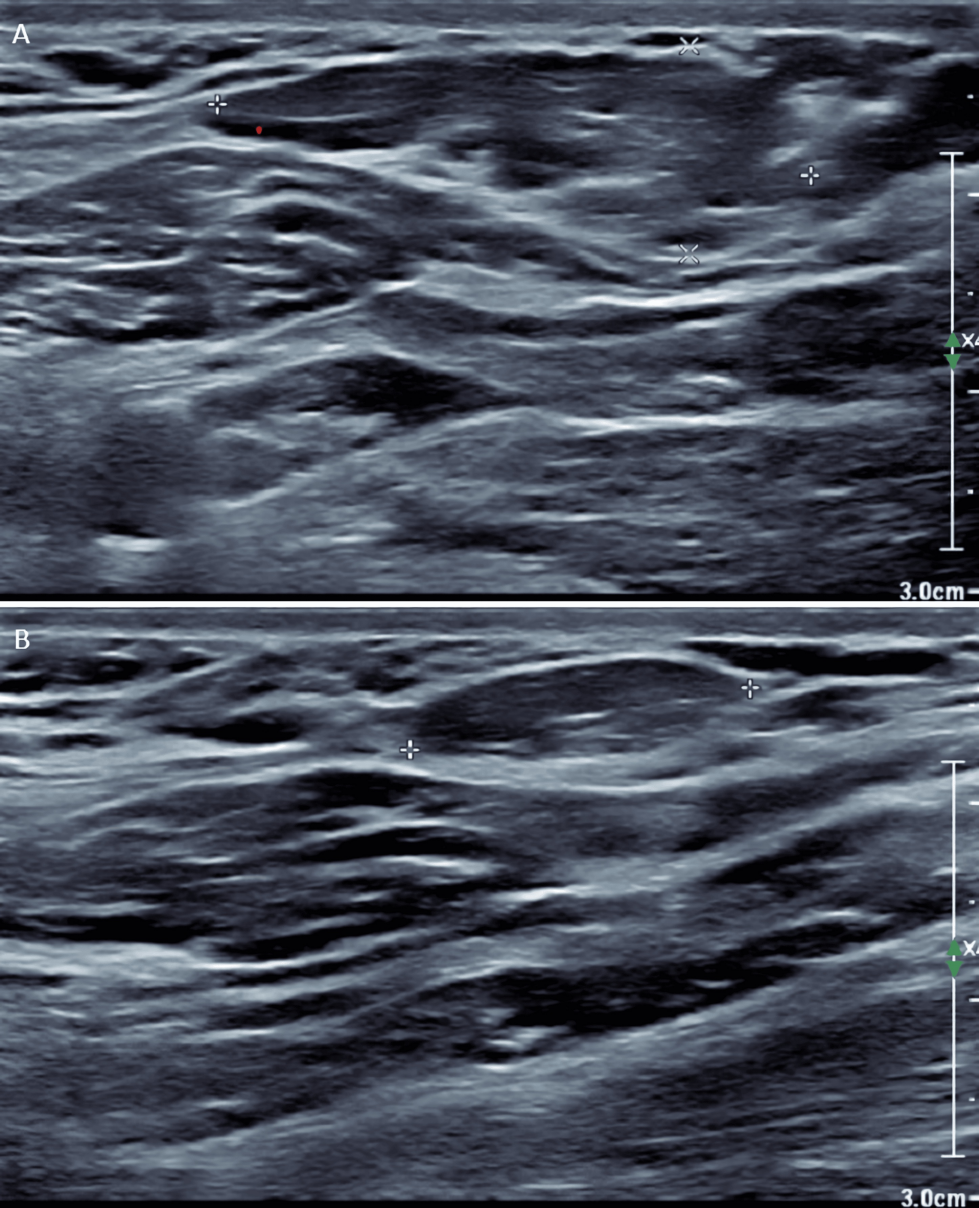
Neck ultrasonography demonstrating sonographic features consistent with a simple lipoma (A) and normal cervical lymph nodes (B).

Initial computed tomography (CT) of the neck identified a poorly defined mass lesion involving the left parapharyngeal and retropharyngeal deep neck space (Figure 1A and Figure 1B). Subsequent ultrasound characterized the mass as a conventional lipoma without concerning features and no evidence of cervical lymphadenopathy (Figure 2). Due to the lack of a regional otolaryngology service, urgent outpatient otolaryngology follow-up was organized for further work-up. Oral cavity inspection and flexible nasal endoscopy demonstrated features consistent with laryngopharyngeal reflux.

**Figure 3:**
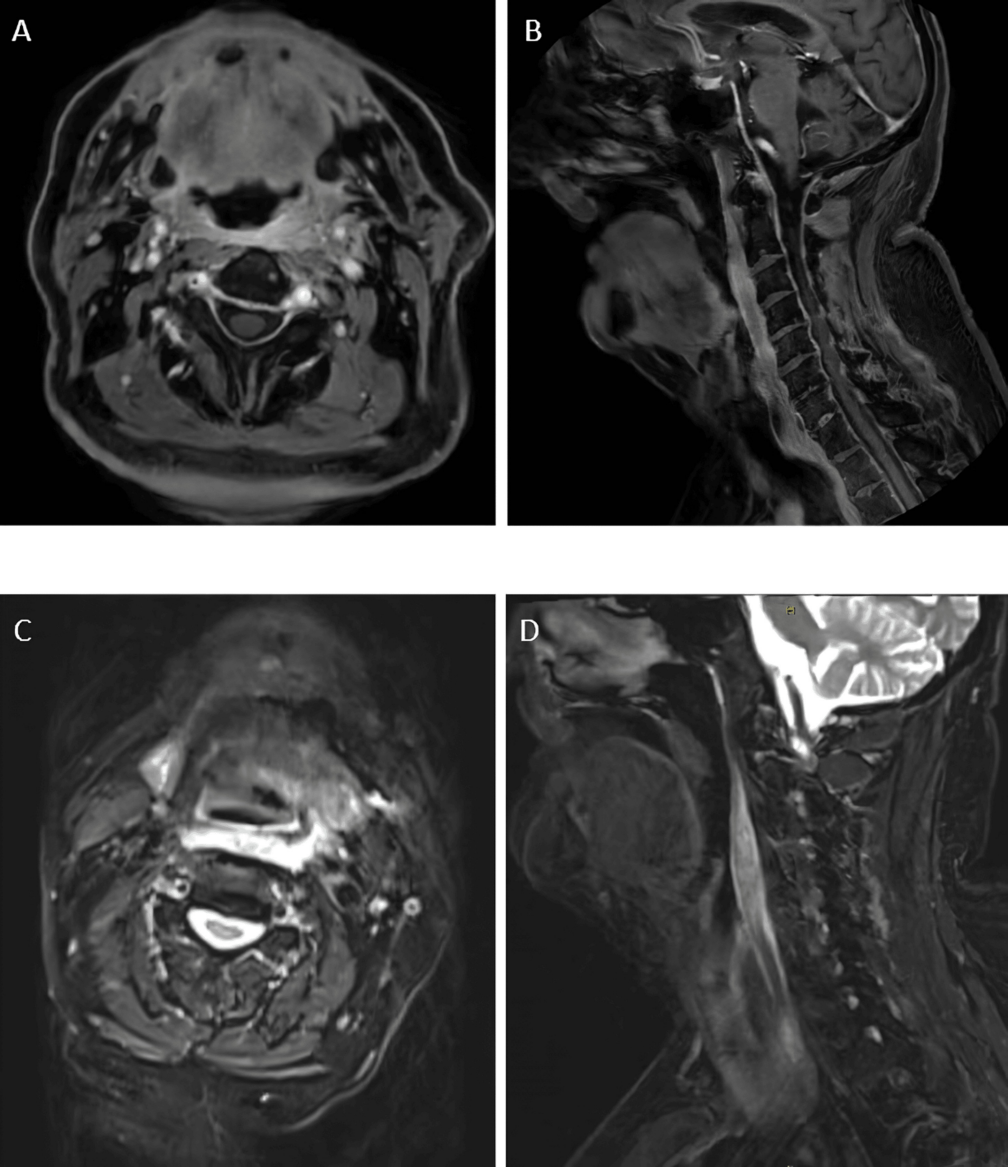
Multi-sequence, multiplanar gadolinium-enhanced images demonstrating inflammatory changes in the left retropharyngeal space with radiological features suggestive of longus colli calcific tendonitis. (A, B) are T1-weighted images showing contrast enhancement in the left retropharyngeal and prevertebral spaces in the axial and sagittal planes, respectively, while (C, D) show concordant edema on T2-weighted sequences.

The patient re-presented to the emergency department twice after discharge, exhibiting signs of systemic inflammatory response syndrome (SIRS) and restricted neck movement. On both occasions, he was transferred to an ENT center. Interval CT (Figure 1C and Figure 1D) and gadolinium-enhanced magnetic resonance imaging (MRI) (Figure 3) indicated a diagnosis more consistent with retropharyngeal effusion secondary to longus colli calcific tendonitis rather than an occult neoplasm.

A medical consultation was requested to establish a unifying diagnosis due to the severity of symptoms and frequent hospital presentations, which seemed disproportionate to uncomplicated longus colli calcific tendonitis. The patient’s blood profile (see Table 1) consistently demonstrated normocytic anemia and lymphopenia with a normal white cell count. Septic screens were unremarkable. Serum biochemistry demonstrated nonspecifically elevated CRP and mildly elevated serum procalcitonin. Serum protein electrophoresis suggested an inflammatory process, and the kappa/lambda free light chain ratio further suggested an inflammatory etiology.

**Table 1:** Pathology results obtained from the patient’s blood work while an inpatient.

	Value	Units	Reference range
Hemoglobin	97	g/L	120-180
Hematocrit	0.31		0.35-0.51
Mean corpuscular volume	90	fL	80-100
Red cell count	3.42	x10^12^/L	3.50-6.00
Platelet count	467	x10^9^/L	140-400
White cell count	7.2	x10^9^/L	3.5-11.0
Lymphocytes	0.53	x10^9^/L	1.00-4.00
C-reactive protein	249	mg/L	<5.0
Procalcitonin	2.59	ug/L	0.50-2.00
Sodium	130	mmol/L	135-145
Potassium	4.1	mmol/L	3.5-5.2
Chloride	98	mmol/L	95-110
Bicarbonate	22	mmol/L	22-32
Urea	13.3	mmol/L	2.9-8.2
Creatinine	130	umol/L	64-108
Albumin	17	g/L	35-50
Globulin	51	g/L	25-45
Alpha 1	7	g/L	3-6
Alpha 2	14	g/L	4-10
Beta	12	g/L	5-11
Gamma	18	g/L	7-18
Kappa/Lambda ratio (N Latex)	0.65		
Lambda free light chains (N Latex)	68	mg/L	8-27
Kappa free light chains (N Latex)	44	mg/L	7-22

**Figure 4:**
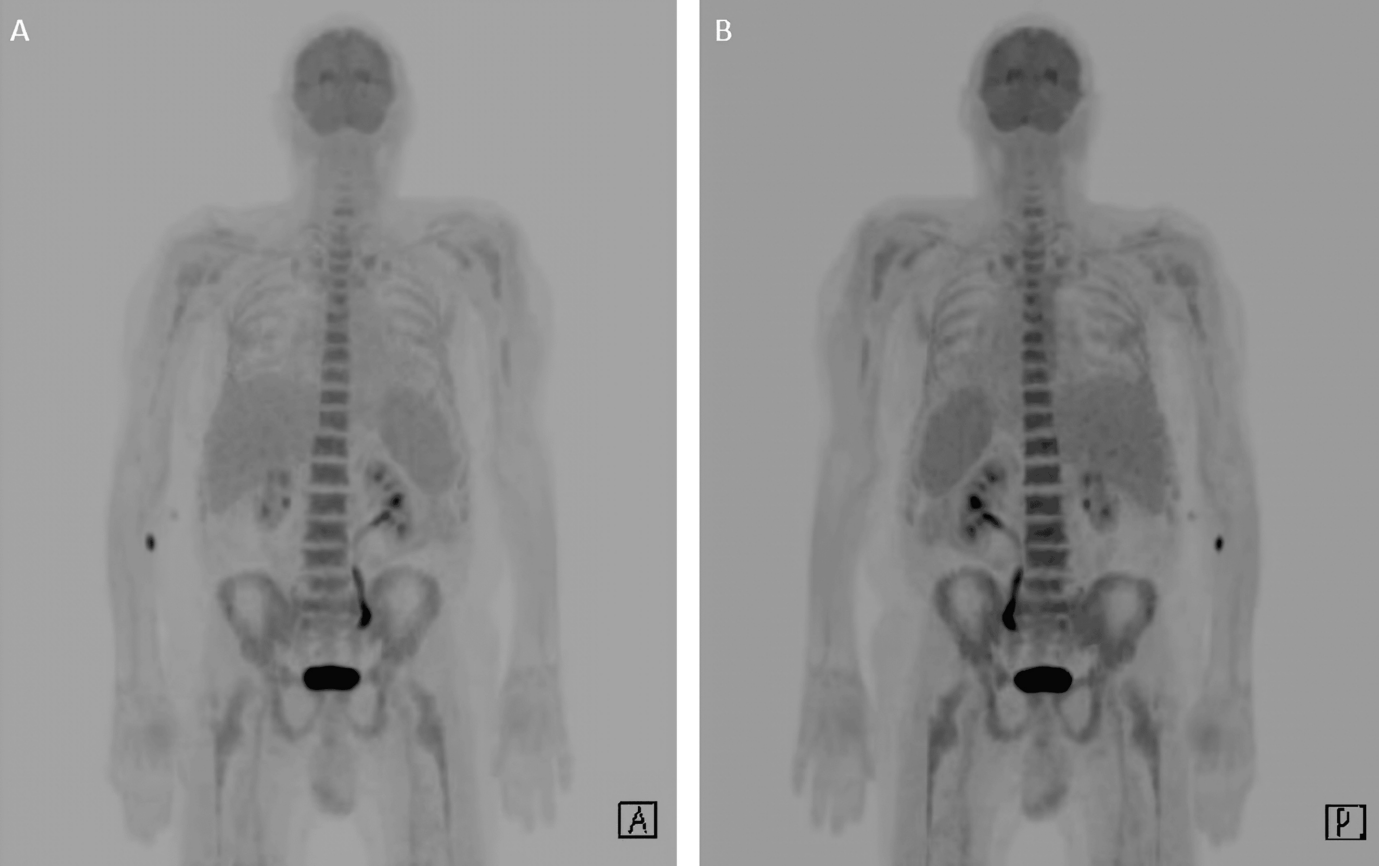
FDG PET-CT MIP series from anterior (A) and posterior (B) perspectives, demonstrating increased uptake within the patient’s bone marrow and spleen. FDG, fluorodeoxyglucose; PET, positron emission tomography; CT, computed tomography; MIP, maximum intensity projection

Fluorodeoxyglucose positron emission tomography-computed tomography (FDG-PET-CT) demonstrated no primary neoplasm but revealed diffuse splenic and bone marrow uptake (Figure 4).

Subsequent bone marrow aspirate was arranged by hematology due to persistent bicytopenia and PET-CT findings; this revealed the presence of vacuoles, raising suspicion for VEXAS syndrome. The diagnosis was confirmed through a gene panel, which identified variants in the UBA1 and DNMT3A genes in the bone marrow aspirate (see Table 2). The UBA1 gene is responsible for producing the E1 enzyme that catalyzes the regulatory protein ubiquitin, found ubiquitously in most tissues, while DNMT3A normally functions as a repressor of inflammation [1,3]. He was referred to immunology for ongoing multidisciplinary care.

**Table 2:** Bone marrow aspirate and trephine results confirming VEXAS syndrome, showing reactive bone marrow without concurrent myelodysplasia. VEXAS, Vacuoles, E1 enzyme, X-linked, autoinflammatory, somatic; VAF, variant allele frequency

Bone marrow aspirate and trephine	Hypercellular marrow with reactive features. Features of anemia of chronic disease present. Mild granulocytic dysplasia. Vacuolated granulocytic precursors present, which is nonspecific
Flow cytometry	No monoclonal B-cell or aberrant T- or NK-cell population detected. There is a plasma cell population comprising 2.6% of the total cell
Supplementary test(s) - molecular	UBA1 mutation defected (VAF 37%) and DNMT3A detected (VAF 19%)

## Discussion

The variable clinical manifestations in VEXAS syndrome pose significant diagnostic challenges to all clinicians, particularly in atypical cases such as this. While ENT manifestations of periorbital or vocal cord edema and auricular chondritis have been reported with VEXAS syndrome, deep neck space involvement has not been previously described [4].

Connective tissue disease is well documented in VEXAS syndrome, predominantly manifesting through tendinitis or periorbital myositis [5]. The likely cause of this patient’s para- and retropharyngeal phlegmon is relapsing longus colli calcific tendinitis as demonstrated on imaging. The patient’s bicytopenia and serum protein electrophoresis were helpful in establishing a non-infectious etiology. FDG PET-CT offered diagnostic clues, demonstrating increased uptake in the spleen and bone marrow, another feature of VEXAS syndrome (Figure 4), while simultaneously excluding an infectious or malignant process in the deep neck space [3,6].

There is currently no consensus on consistently effective therapies for treating VEXAS syndrome [7]. Although this patient initially responded well to glucocorticoid treatment, attempts to wean resulted in symptom relapse and hospital re-presentations. This issue is well documented and highlights the need for further research into alternative long-term treatment modalities [8].

VEXAS syndrome is characterized by a somatic mutation in the ubiquitylation UBA1 gene in blood cell precursors, which drives the disease process. The most frequently reported UBA1 gene mutation involves methionine at amino acid 41. This patient’s gene panel identified the variant p.Met41Val(c.121A > G), which is thought to portend more aggressive disease and a poorer prognosis, consistent with his presentation and relapse pattern [2,3].

Other somatic mutations can coexist with UBA1, such as the DNMT3A mutation found in this patient (c.1792C>T, p.(Arg598*)). This coexisting mutation occurs in approximately 9.2-22% of cases and is thought to contribute to inflammation by providing a competitive advantage to the mutated UBA1 cells [3].

Currently, features such as fever, skin manifestations, and pulmonary infiltrates are associated with worse prognosis, poorer response to drugs, and higher mortality, compared with those affected by relapsing polychondritis, the most reported ENT manifestation thus far [1,9]. At present, the impact of deep neck space involvement on clinical outcomes remains unknown, but only through more reported cases can clinicians develop an understanding and awareness of the manifestations of this rare condition. This case report aims to raise awareness of deep neck space presentation in VEXAS syndrome and to guide management. Additionally, it calls for further investigation of specific mutant genotypes and mutations related to ENT presentations.

## Conclusions

We report the first case of VEXAS syndrome presenting as relapsing deep neck space involvement, hypothesized to be secondary to longus colli calcific tendinitis and cytokine storm. Despite many VEXAS syndrome patients displaying cutaneous and/or pulmonary disease, this patient presented without these features. More frequent ENT presentations, including relapsing polychondritis, were also absent.

This case highlights a rare but important differential to consider in patients with unexplained relapsing systemic inflammation and atypical inflammatory deep neck pathology, even in the absence of more commonly described VEXAS features, to reduce diagnostic delay. Given the syndrome's complex multi-system involvement, a multidisciplinary approach and effective communication across teams are vital for accurate and timely diagnosis. The diverse presentations of this syndrome appear to influence prognosis, and additional case reports may help clarify the impact of ENT involvement.
